# Congenital paraesophageal hiatus hernia with gastric volvulus

**DOI:** 10.4103/0971-9261.42574

**Published:** 2008

**Authors:** Ashok Y. Kshirsagar, S. L. Shinde, M. D. Ahire, Y. B. Langade

**Affiliations:** Department of Surgery, Krishna Institute of Medical Sciences, Deemed University, Karad, Satara, Maharashtra, India

**Keywords:** Congenital paraesophageal hiatus hernia, gastric volvulus

## Abstract

Paraesophageal hiatus hernia is rarely seen in the neonatal period. An intrathoracic gastric volvulus complicating such a hernia is rarer. The upper gastrointestinal tract contrast study is diagnostic. Rapid diagnosis and treatment is essential. It avoids lethal complications as gastric dilatation, gangrene and perforation, which in turn may lead to cardiopulmonary arrest.

## INTRODUCTION

Congenital paraesophageal hiatus hernia with gastric volvulus is uncommon entity in neonate characterized by an abnormal degree of rotation and migration of the stomach into the thoracic cavity through an abnormally wide esophageal hiatus with the gastroesphageal junction in its normal intraabdominal position. It can be confused with esophageal atresia, duodenal atresia, tracheoesophageal fistula and right diaphragmatic hernia. Early diagnosis and prompt surgical treatment is required. We report a case of a 10-day-old neonate having congenital paraesophageal hiatus hernia with gastric volvulus, which was successfully treated surgically.

## CASE REPORT

A 10-day-old male neonate weighing around 3 kg presented with minimal respiratory distress with persistent non-bilious vomiting since birth. On examination, there was tachypnea and tachycardia with dehydration and mild fullness in the epigastric region.

**Figure 1:**
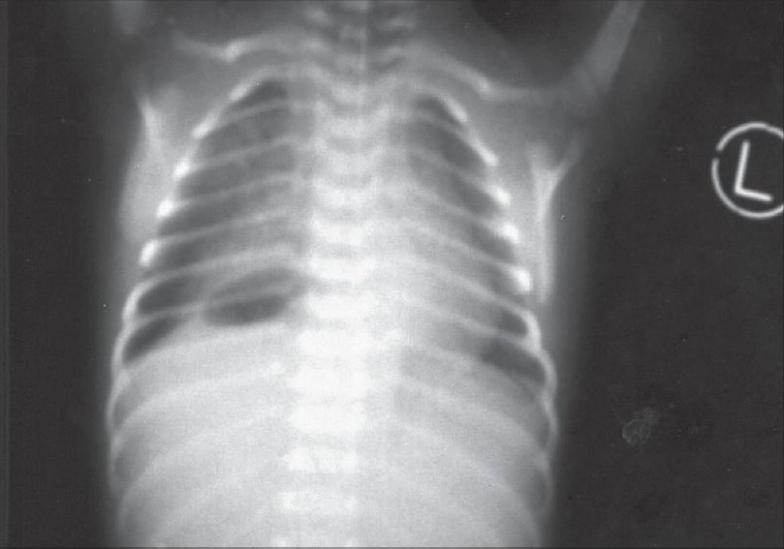
Plane X-ray chest showing the presence of abnormal gas shadow in the right paravertebral region in the lower chest

**Figure 2:**
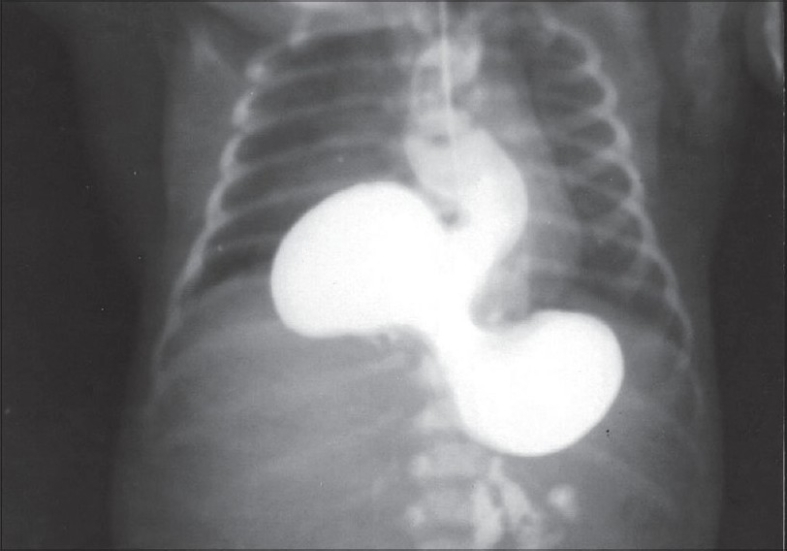
The contrast study showing dilated esophagus with stomach in state of volvulus

Plain X-ray of the chest showed the presence of an abnormal gas shadow in the right paravertebral region in the lower chest [Figure 1]. Ryle's tube was passed and it drained non-bilious aspirate. Barium contrast study revealed the dilatation of the esophagus with stomach in the right side of thorax in a state of volvulus with some contrast passing distally [Figure 2].

**Figure 3:**
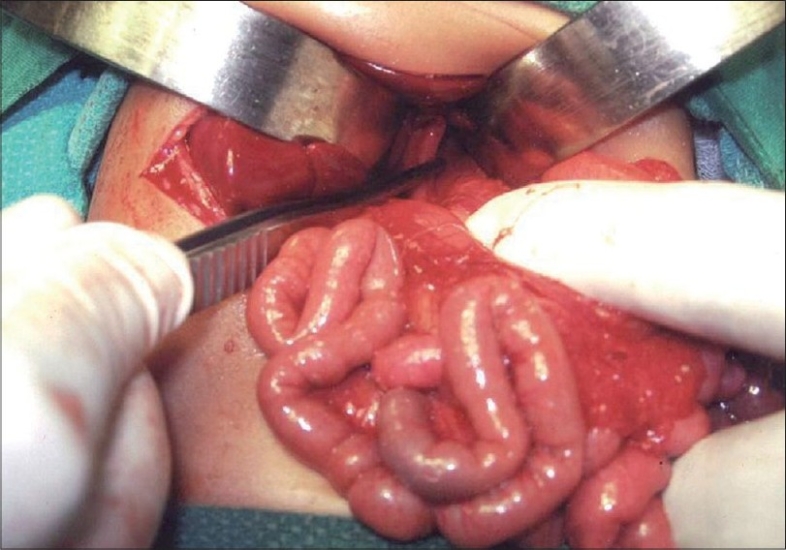
Intraoperative picture showing the herniation of stomach through an enlarged esophageal hiatus

After the correction of dehydration, the patient was taken for an emergency surgery. On exploration, the stomach was not found in the abdominal cavity and the gastroesophageal junction was at its normal site (Figure 3). Further exploration confirmed the presence of the entire stomach within the chest cavity. Herniation of the stomach had occurred in the midline through an enlarged esophageal hiatus with the stomach in a state of mesenterioaxial volvulus. The gastric volvulus was reduced followed by the repair of the hiatal defect with gastropexy. The infant recovered well.

## DISCUSSION

Paraesophageal hiatus hernia accounts for approximately 5% of all the diaphragmatic hernias in childhood.[1] However, a congenital paraesophageal hiatus hernia with a complete or partial right intrathoracic gastric volvulus has been rarely reported. There is an anatomical defect in the hiatus without any derangement of the gastroesophageal sphincter mechanism.

Most of the congenital paraesophageal hiatus hernias occur sporadically; however, there are case reports of familial occurrence, including a report of two affected siblings.[2]

Clinical presentation depends on the degree of rotation and obstruction with the vascular compromise of stomach. There may be vomiting after meals, symptoms of obstruction, collapse and cardiopulmonary arrest.[3] Sawaguchi has attributed vomiting in young infants to the maldevelopment of hiatal function.[4] In gastric volvulus, severe epigastric pain and distention, violent unproductive retching and inability to pass nasogastric tube comprise the classical triad. Chest X-ray shows an abnormal gas shadow in the right paravertebral region in the lower chest and contrast study is diagnostic.

Acute gastric volvulus is a surgical emergency and suspicion is the key to successful management. Delay in recognition can cause strangulation and perforation due to vascular compromise. Untwisting of gastric volvulus with the repair of hiatal defect with gastropexy is a satisfactory solution to this life-threatening condition. However, laparoscopic gastropexy has also been reported.[5]

Although a rare condition, when a neonate is presenting with tachypnea, vomiting and abdominal distention, the possibility of paraesophageal hiatus hernia with gastric volvulus should always be considered. This case highlights the fact that a clinicoradiological correlation is necessary for arriving at an accurate diagnosis and speedy recovery.
